# Using PACS for teaching radiology to undergraduate medical students

**DOI:** 10.1186/s12909-024-05919-9

**Published:** 2024-08-28

**Authors:** Mojtahedzadeh Rita, Mohammadi Aeen, Farnood Rajabzadeh, Akhlaghi Saeed

**Affiliations:** 1 Department of e-Learning in Medical Education, School of Medicine, Center of Excellence for E- learning in Medical Education, Tehran University of Medical Sciences, Tehran, Iran; 2 Department of Radiology, Faculty of Medicine, Mashhad Medical Sciences, Islamic Azad university, Mashhad, Iran; 3 Department of Community Medicine, University of Medical Sciences, Mashhad, Mashhad, Iran

**Keywords:** Radiology education, PACS, Medical students

## Abstract

**Background:**

Traditional radiology education for medical students predominantly uses textbooks, PowerPoint files, and hard-copy radiographic images, which often lack student interaction. PACS (Picture Archiving and Communication System) is a crucial tool for radiologists in viewing and reporting images, but its use in medical student training remains limited.

**Objective:**

This study investigates the effectiveness of using PACS (Picture Archiving and Communication System) for teaching radiology to undergraduate medical students compared to traditional methods.

**Methods:**

Fifty-three medical students were divided into a control group (25 students) receiving traditional slide-based training and an intervention group (28 students) using PACS software to view complete patient images. Pre- and post-course tests and satisfaction surveys were conducted for both groups, along with self-evaluation by the intervention group. The validity and reliability of the assessment tools were confirmed through expert review and pilot testing.

**Results:**

No significant difference was found between the control and intervention groups regarding, gender, age, and GPA. Final multiple-choice test scores were similar (intervention: 10.89 ± 2.9; control: 10.76 ± 3.5; *p* = 0.883). However, the intervention group demonstrated significantly higher improvement in the short answer test for image interpretation (intervention: 8.8 ± 2.28; control: 5.35 ± 2.39; *p* = 0.001). Satisfaction with the learning method did not significantly differ between groups (intervention: 36.54 ± 5.87; control: 39.44 ± 7.76; *p* = 0.129). The intervention group reported high familiarity with PACS capabilities (75%), CT principles (71.4%), interpretation (64.3%), appropriate window selection (75%), and anatomical relationships (85.7%).

**Conclusion:**

PACS-based training enhances medical students’ diagnostic and analytical skills in radiology. Further research with larger sample sizes and robust assessment methods is recommended to confirm and expand upon theses results

**Supplementary Information:**

The online version contains supplementary material available at 10.1186/s12909-024-05919-9.

## Introduction

Radiology is a fundamental component in basic medical education, bridging the gap between anatomy and clinical practice. Like other fields of medical education, radiology education faces the challenge of transitioning from passive learning to interactive and experiential learning [1, 2]. With the expansion of the field of radiology, radiology education has undergone a revolution. Doctors used to carry plain films and show them using projectors or view boxes because plain films were the only main diagnostic method in radiology during the 1970s. Since the introduction of computed tomography (CT) and magnetic resonance imaging (MRI) in the late 1980s, the increase in the amount of image data associated with these imaging modalities has led to a greater demand for compatible information storage systems. Therefore, the picture archiving and communication system (PACS), capable of storing, retrieving, distributing, analyzing, and digitally processing medical images, has become an essential tool in clinical work today [3–5]. However, due to hardware and software limitations, the use of PACS in radiology education remains somewhat limited [6, 7]. Currently, most radiology education still relies heavily on textbooks and traditional computer media such as PowerPoint or Word files both of which lack student interaction. PACS offers advantages such as interactive image viewing, 3D reconstruction capabilities, and the ability to simulate real-life radiology practice, which traditional methods lack. These features enhance students’ understanding and interpretation of radiological images, addressing the shortcomings of conventional methods. There is a minimal probability for a medical student to see whole images like a real radiologist in class. It is often a challenge for them to understand 3D anatomical images, as well as a comprehensive view of diseases. Consequently, some students may attempt to independently identify abnormal findings and analyze and formulate radiological diagnoses. According to one study, only a limited number of final-year medical students had satisfactory basic radiology interpretation skills, which necessitates the search for a more effective method of training [8].

Recent advancements in radiology teaching methods have previously been reported in addition to face-to-face teaching, including problem-based learning (discussion of a case or scenario consistent with curriculum objectives and students’ independent research to complete subject knowledge and share findings), case-based learning (showing several radiographs of the same subject and discussing them), and team-based learning (student collaboration by creating learning groups) [8].

In contrast to these conventional methods, a new method was created under the concept of learning from experience. This virtual method is based on individual learning in the PACS software environment, enabling students in the role of radiologists to interpret and diagnose radiology in a simulation environment. All common items are shown to the student using PACS instead of selected specific images. Students are allowed to see the whole image, do basic reconstructions of the images freely, and find specific features of the image by themselves. During this process, students can access PACS and clinical information, integrating clinical knowledge and 3D reconstruction ability, essential to arriving at radiological diagnoses PACS enables efficient archiving and transfer of medical images. Initially developed in the U.S. in the 1980s, it later expanded to Europe and Asia, including China, Japan, and Korea [9]. Iran has also implemented PACS, improving its medical imaging infrastructure with global DICOM standards.

The goal of this learning method was to compare the effectiveness of practical radiology training through traditional face-to-face interactive lectures with the virtual practical radiology training method based on individual learning in the PACS software environment for medical students.

The use of PACS in healthcare in Iran has only recently become widespread, primarily for patient management and diagnosis, and is rarely used for educational purposes. Iran, as a country with a rapidly developing healthcare system, faces unique challenges in medical education. This study seeks to compare radiology education in Iran with existing literature and to understand its context in relation to the region and worldwide. Managing medical education effectively is a significant challenge. And this research addresses this by introducing innovative teaching methods. Specifically, current study investigates the effectiveness of using PACS on medical students radiology education compared to traditional methods.

## Methods

### Subjects

The research population was the medical students of the Islamic Azad University of Mashhad during the academic year 2021–2022. The entry criteria were: being a medical trainee student, consent to enter the study, and the exclusion criteria were: students who had previously graduated in radiology or other medical sciences and students who had renewed their course in radiology. participation in the study was voluntary, and students were informed that it would not impact their end-of-section evaluation After obtaining informed consent, they participated in the study. Ethical approval for this study was obtained from the Virtual University of medical sciences with the reference number [IR.VUMS.REC.1400.022]. This proposal was implemented after being approved by the ethics committee and obtaining the code of ethics.

### Participants

The sample size was calculated using power analysis to ensure the study had sufficient power to detect a statistically significant difference between the control and intervention groups. Assuming an effect size of 0.5, a significance level (alpha) of 0.05, and a power of 0.80, it was determined that at least 50 participants were needed. To account for potential dropouts and ensure robustness, a total of 53 students were included in the study. According to the calculated sample size, four rotations of radiology internship students were included in the study for each of the control and intervention groups (each rotation is about 5–10 students). Due to the prevention of contamination, the first four rotations were assigned to the control group and the next four rotations to the intervention group.

The validity of the tools used in this study was established through expert review and pilot testing. Content validity was confirmed by 10 faculty members specializing in radiology. Reliability was assessed using Cronbach’s Alpha, yielding a coefficient of 0.91, indicating high internal consistency. In this study, three tools were used: measuring the level of knowledge, measuring the level of performance, and measuring the satisfaction of students in both groups (Appendix 1) and self-evaluation for PACS learning in the intervention group (Appendix 2). After one month of class, the final exam was taken which was a combination of 20 multiple choice questions and 5 short answer type questions (description and image recognition). The scores of the questions were collected as an objective assessment. To provide a subjective assessment of radiology learning, all students were invited to complete a satisfaction questionnaire on how radiology was taught. Also, the students of the intervention group were invited to complete a questionnaire for their self-evaluation of the amount of PACS learning. A 5-point Likert scale was used in both researcher-made questionnaires. The questionnaire used was created for this study. Informed consent was obtained from each patient whose data was used in the study, ensuring they were fully aware of how their medical images would be utilized for educational purposes.

### Familiarization with PACS

Before starting the study with the PACS system, students were given an introductory session that covered the basics of PACS functionality, including how to navigate the software, view and manipulate images, and use the various tools available for image analysis.

### Knowledge and performance measurement tools

In the knowledge section, questions evaluated theoretical content, and the performance section involved diagnosing radiographic image. Students described the type of radiography, pathological signs, and the final diagnosis. Multiple-choice questions and short answer questions were used to assess knowledge and performance The specific type of radiography used in this study included plain radiographs, computed tomography (CT) scans, and magnetic resonance imaging (MRI). These imaging modalities were chosen to cover a broad spectrum of radiological techniques relevant to the medical curriculum. In the subject of knowledge, 20 multiple-choice questions were proposed based on the objectives of the lesson and the blueprint, which was approved by two colleagues of the radiology department, which must have been consistent with the objectives of the lesson. In the discussion of the performance of 5 of radiology images, which again corresponded to the objectives of the lesson and the blueprint, and it was approved by two colleagues of the radiology department that the objectives of the lesson were covered, they were provided to the students, and the students had to describe and diagnose the radiographies. The radiology images in both groups adequately covered the goals, but they were taught to the students in two different ways described.

### Student satisfaction questionnaire

This questionnaire aimed to determine student’s satisfaction with the educational method. It consisted of ten questions graded on a 5-point Likert scale the range of scores was between 10 and 50 and higher scores indicating greater satisfaction. The content and form validity were confirmed by 10 faculty members and reliability was obtained by Cronbach’s Alpha test of 0.91.

### Student self-assessment questionnaire

This questionnaire evaluated the learning rate of the PACS teaching method. It consisted of twelve questions graded on a 5-point Likert scale, and the range of scores was between 12 and 60, and higher scores indicate learning. Content and form validity were confirmed by 10 faculty members and reliability was assessed with a Cronbach’s Alpha of 0.91.

### Implementation method in the control group

The teaching strategy involved traditional face-to-face interactive lectures using PowerPoint presentations. The practical part included demonstrating selected radiographic images on slides and discussing their interpretation.

This method aimed to develop the student’s ability to diagnose and interpret radiographs through structured lectures and guided discussions. A pre-test was conducted in the first session to determine the student’s initial knowledge and performance levels. The classes were held daily in person. After teaching the theoretical part with a PowerPoint presentation, radiographic images were shown to the control group for interpretation and discussion. This conventional method aimed to develop the ability to diagnose and interpret radiographs. The post-test to determine knowledge and performance was performed and the education satisfaction questionnaire was completed at the end of each rotation.

Bias caused by human factors during the teaching of the two groups was controlled by standardizing the teaching materials and methods across both groups. Additionally, the instructors were blinded to the group assignments to prevent any conscious or unconscious bias in teaching and assessment.

### Implementation method in the intervention group

The stages of developing the training course using PACS software and DICOM were as follows: 1). Initial planning and curriculum alignment, 2) Selection of relevant radiographic cases, 3) Configuration of PACS workstations, 4) Training faculty on PACS software, and 5) Implementation of PACS-based learning sessions for students, followed by assessment and feedback.

After the control group, the rotations of the intervention group were included in the study, and the pre-test was administered to the students of the intervention group. Assessment of knowledge with multiple choice questions and performance with radiographic images was with short answer questions. The classes were held daily in person. In the intervention group, after participating in the theoretical part of the course, which was similar to the control group and was held face-to-face, for the practical part, they were trained in a virtual way with Adobe Connect software, and there was no face-to-face class for radiography images. In this way, students were given access to PACS Radiant software (installation on personal desktop). Following the teaching of the theoretical part, based on the goals of the radiology course for medical trainees, a number of images of the brain, lungs, bones, urinary tract, and digestive system (including radiography, CT and MRI) were assigned to the students of the intervention group, and the images of these patients were completely at their disposal.

The computers used were personal desktops with standardized configurations. Adjustments and calibrations were made to ensure all students could view images with consistent quality and brightness, replicating the clinical environment as closely as possible. This software enables students to perform basic operations with images, such as windowing, comparing different MRI sequences, and performing cross-sectional reconstruction (MPR) or 3D reconstruction, exactly as a radiologist does and has the facilities. After studying the material and checking the images, the students were required to announce the completion of their study to the teacher and they were given the opportunity to review the pictures, ask questions, and solve problems with the teacher in the virtual space.

The post-test to determine knowledge and performance was performed in the intervention group. The education satisfaction questionnaire was completed at the end of each rotation. The self-assessment questionnaire for PACS learning was completed at the end of each rotation.

### Data analysis

The data was analyzed with SPSS-17 software, IBM, US. Central and dispersion indices were used in the descriptive statistics report, and a T-test was used in the analytical section, independent t-test, paired t-test and, chi-square test were used to compare the data. The confidence level was set at *p* < 0.05.

## Results

A total of 52 students entered this study, 28 students in the intervention group and 25 in the control group. The students were similar in terms of age, gender, and overall academic average (*p* = 0.05) (Table 1). The average age in the control group is 26.04 ± 3.96 and in the intervention group is 24.29 ± 2.14. The result of the independent t-test shows that the average age in the two groups is not different (*P* = 0.060). The average overall academic grade point average of the medical course in the control group is 15.73 and in the intervention group is 16.01, which has no difference (*P* = 0.383) (Table 1).

The control group included 25 people, 16 of whom were women and 9 of whom were men, and the intervention group included 28 people of whom 16 were women and 12 were men. The result of the chi-square test shows that the two groups do not differ in terms of gender (*P* = 0.610). Evaluation result: At the beginning of the exam, there were two parts of a multiple-choice test and a short answer for the interpretation of radiology images (pre-test). The same exam was done twice at the end of the one-month session (post-test). It was a multiple-choice test to check knowledge and a short answer test to check performance.

The result of the independent t-test shows that the score of the multiple-choice test before and after the intervention, as well as the changes in the test score, are not different in the two groups. (*P* = 0.084, *P* = 0.883, *P* = 0.764) The result of the paired t-test shows that the multiple-choice test scores of the students before and after the intervention differ between the case and control groups, and it is higher after the intervention. (*P* < 0.001, *P* < 0.001) (Table 2) The result of the independent t-test shows that the score of the student’s short answer test, which was for the interpretation of radiology images, is not different before and after the intervention (*P* = 0.002 and *P* = 0.444, respectively). The changes in the test scores are different in the two groups and are more in the intervention group. (*P* < 0.001) The result of the paired t-test shows that the score of the short answer test of the students before and after the intervention is different according to the case and control groups, and it is higher after the intervention. (*P* < 0.001, *P* < 0.001)

The result of the independent t-test shows that there is no difference in the level of satisfaction with the teaching method between the two control groups with a score of 39.44 ± 7.76 and the intervention group with a score of 36.54 ± 5. (*P* = 0.129) (Table 3).

The analysis of the satisfaction questionnaire in the intervention group showed that most students were satisfied with the organization (64%) and interaction of the learning activity (64%) (Table 3). Most students use this learning activity to learn radiology (85%). They found it useful. More importantly, a large percentage of students stated that PACS training encouraged personal interest in radiology (82%) as well as satisfaction with the quality of learning (71%). Also, in the intervention group, based on the self-evaluation form, they stated that with the abilities of PACS (75%), the principles of CT (71.4%) and its interpretation (64.3%), choosing the appropriate window (75%), the location of different organs in the image (82.9%) and their vicinity (85.7%) are familiar (Table 3). An evaluation of the impact of the intervention on participants’ knowledge is included, showing significant improvements in their understanding and diagnostic skills, highlighting the effectiveness of the PACS-based training method.

**Table 1:** Demographic information

Variable	Control Group (*n* = 25)	Intervention Group (*n* = 28)	*P*-value
Age (mean ± SD)	26.04 ± 3.96	24.29 ± 2.14	0.060
GPA (mean ± SD)	15.73 ± 1.31	16.01 ± 1.03	0.383
Female (n, %)	16 (64%)	16 (57.1%)	0.610
Male (n, %)	9 (36%)	12 (42.9%)	-

**Table 2:** Multiple-choice and short answer question scores

Variable	Control Group (*n* = 25)	Intervention Group (*n* = 28)	*P*-value
Multiple-choice Questions			
Pretest Score (mean ± SD)	1.87 ± 4.89	1.5 ± 4.01	0.084
Posttest Score (mean ± SD)	3.59 ± 10.76	2.94 ± 10.89	0.883
Score Difference (mean ± SD)	4.3 ± 6.48	3.26 ± 7.16	0.764
Short Answer Questions			
Pretest Score (mean ± SD)	3.19 ± 6.79	2.16 ± 4.02	0.002
Posttest Score (mean ± SD)	2.53 ± 11.66	2.12 ± 12.16	0.444
Score Difference (mean ± SD)	2.39 ± 5.35	2.28 ± 8.8	0.001

**Table 3:** Satisfaction scores

Variable	Control Group (*n* = 25)	Intervention Group (*n* = 28)	*P*-value
Score of Satisfaction (max 50)	39.44 ± 7.76	36.54 ± 5.87	0.129

## Discussion

Traditional practical radiology training that continues to be used today provides only a cross-section of the entire routine imaging. While this teaching method may be useful in helping students manage the features of routine imaging, it may be inadequate for learning anatomy [10]. Hence, students may have difficulty interpreting images independently during clinical practice when they are expected to do so [11]. Although a variety of radiology educational models such as problem-based learning and the use of dynamic images can solve part of this problem, images of the main workplace are the most ideal learning method [12, 13]. The experiential learning theory, developed by Dewey, Kolb and others provide explanations for how students learn things in their own way as they react to their perceptions of a real experiences. This concept is explained by principle of constructionism, which is the base of experiential learning [13].

During this study, a training course using PACS software and DICOM viewer was developed to simulate a work environment that reflects the typical clinical work of a radiologist. The results of the study indicated that this educational approach allows for better clinical guidance, which is necessary to help students form a holistic view of anatomy and pathology. Most importantly, this educational method helps students to develop critical thinking and a systematic approach to formulating imaging interpretation and differential diagnosis, which may be partially due to the exploratory atmosphere of the experiential learning mode. Apart from the objective improvement in imaging descriptions and interpretations, subjective improvements in self-confidence from students’ feedback to self-assessment questionnaires, as well as skills including determining the order of imaging reading, choosing the appropriate window, and also choosing the reconstruction method, which may result under the influence of direct activity during The course of learning and discussion should be free. In addition, the experiential approach allows for better interactions that increase interest in radiology [14].

To provide students with access to the Radiant PACS software (installed on their personal desktops), following the theoretical section and based on the objectives of the radiology course for medical trainees, a number of images from the brain, lungs, bones, urinary, and gastrointestinal systems (including radiography, CT, MRI) were assigned to the intervention group. These patient images were fully available to them. This software enables students to perform basic operations on images, such as window adjustment, comparing different MRI sequences, and performing multiplanar reconstruction (MPR) or 3D reconstruction, exactly as a radiologist does within the PACS system.

To resolve the issue of patient confidentiality, all patient identifiers were removed from the images before they were made accessible to students. Additionally, access to PACS was restricted to ensure that students could only view and analyze the images without accessing sensitive patient information.

Undergraduate students had limited access to PACS, ensuring they could not modify or delete any content. Additional software controls were implemented to restrict access and prevent any unauthorized changes. This ensured that the integrity of the medical images was maintained, and patient care data was not compromised.

Our study shows the effectiveness of PACS in training in the study of anatomical imaging. Anatomy is the basis of radiology training. In theory, reading CT and MRI images is a good way to study anatomy because continuous scanning helps students understand the three-dimensional concepts of the relative adjacencies of body parts [15, 16]. Globally, they concluded that anatomical imaging increases the quality and efficiency of teaching human anatomy [17]. However, it is difficult to discern the entire anatomical structure from a single cross-section of the image, which increases students’ confusion [16]. The results of this study provide evidence that continuous scan reading improves students’ comprehensive understanding of anatomy. Furthermore, by using multiple reconstruction methods, 3D images are more comprehensively examined by students, which has been confirmed by other studies [18].

The integration of PACS in medical education has been shown to enhance the learning experience by providing students with interactive and practical tools for understanding radiological images. Recent advancements in healthcare technology acceptance highlight the importance of user-friendly interfaces and training for successful implementation [19]. Moreover, the current state of medical education in the UK emphasizes the adoption of advanced technologies like PACS to improve educational outcomes and prepare students for real-world clinical environments [20]. The utilization of big data technologies in conjunction with PACS further enhances the management and analysis of medical images, facilitating a more personalized and effective learning experience for medical students [21]. Additionally, recent market reports indicate a steady growth in the adoption of medical imaging technologies, including PACS, driven by advancements in AI and machine learning, which are poised to revolutionize medical education [22]. These developments collectively underscore the critical role of PACS in modernizing medical education and improving the quality of training for future healthcare professionals. Also, the implementation of PACS could significantly enhance radiology education by providing access to digital imaging resources that may otherwise be unavailable.

Compared to Chen et al.‘s study [1], the study was conducted on 101 students, but our study was on 52 students. Satisfaction with PACS training in Chen’s study was on average 80% and in our study, it was about 65%. The percentage of being interested in radiology in this study and Chen’s study was almost similar. Also, in our study, similar to Chen’s study, there was no difference in pre-test scores between the two intervention and control groups. Also, the final scores in Chen’s study and our study were not significantly different, but the scores of interpretations of pictures, which in our study were equivalent to a number of stereotypes in the form of PowerPoint with short answer questions, showed a significant difference in both our study and Chen’s study.

​ In the study of Restauri [6] and Soman [23], as in our study, PACS was used to teach medical students, and at the end of the course, only a survey form was filled by the students, and the impact of using PACS on the ability to interpret radiology images by students was not done. In the above two studies, after using PACS, students stated that they gained more confidence on interpreting images and would use PACS in the future, which was similar to the survey results in our study. It takes a lot of effort to do this kind of training. PACS and a suitable DICOM viewer represent basic software requirements for training and to protect patient privacy, DICOM data from PACS rather than linking to the original PACS. Copied In this way, a PACS simulation for medical education was obtained [6]. In addition, teacher guidance is a vital element in education. A minimum of 3 instructors with experience in standard radiology training is required for a class, as team discussion is a major component of the training. In experimental courses, students need educational help both to guide reading the picture and to answer the questions. Therefore, teaching professors need specific work experience in the radiology department. Having said that, the lack of a radiology professor prevents the use of this training and this training model acts as a limitation on a larger scale. There are several limitations to the study. First, due to the limited number of supervisors, the sample size was correspondingly limited. Secondly, it was a single study center. Thirdly, due to the limitation of the operation, some students did not answer some of the questions in the questionnaire. Although the probability is very low, it still has the chance to bias the result. Fourth, although we control for faculty and teaching standards between the two groups, human bias is still a factor that cannot be completely avoided in practice. Fifth, although we used objective assessment measures, the study also revealed the weakness of our assessment system in radiology education. The study instrument consisted of paper and pencil tests, with most questions consisting of objective items that test memory, such as multiple-choice questions and short answer questions. Furthermore, the mental items used to test application ability are limited. As a result, only a small part of the final test reflects the difference between the experimental training group and the control group. Other test forms such as bedside examinations and multi-station examinations should be used in the future for better evaluation [24, 25]. In this study, according to the curriculum, students entered the radiology department with different numbers during different periods, and 4 periods of students were entered into the study for each group. The exams were held at the end of the one-month section, so the exam was held in the control group and in the intervention group at different times, although we tried to make the questions the same in terms of number and content similarity. In the study of Chen et al [8], the test was conducted at the end of the semester and simultaneously for two groups. If this study is conducted with a larger number of students and in multiple centers, the results will be more valid.

## Conclusion

PACS-based training is beneficial for medical students, enhancing their diagnostic and analytical skills in radiology. Further research with larger sample sizes and robust assessment methods is recommended to confirm and expand upon theses results. We believe that our findings suggest that PACS which is used routinely in healthcare diagnostic context, can also be used in medical students’ education and healthcare can be integrated in education.

### Electronic supplementary material

Below is the link to the electronic supplementary material.

Supplementary Material 1

Supplementary Material 2

## Data Availability

The demographic and clinical datasets generated and/or analyzed during the current study are available from the corresponding author (Dr. Farnood Rajabzadeh ) upon reasonable request.

## Abbreviations

PACS: Picture Archiving and Communication System
CT: Computed Tomography
MRI: Magnetic Resonance Imaging
GPA: Grade Point Average
DICOM: Digital Imaging and Communications in Medicine
SPSS: Statistical Package for the Social Sciences
MPR: Multi planar Reconstruction
AI: Artificial Intelligence
