# Supplementary material for: Using PACS for teaching radiology to undergraduate medical students
Source: BMC Med Educ. 2024 Aug 28;24:935. doi: 10.1186/s12909-024-05919-9 (PMC11351050; doi:10.1186/s12909-024-05919-9)
Supplement: Supplementary file 1 — Supplementary Material 1 [file 12909_2024_5919_MOESM1_ESM.docx]

| I strongly disagree  (1) | I disagree  (2) | I have no opinion (3) | I agree (4) | I strongly agree (5) |  |
| --- | --- | --- | --- | --- | --- |
|  |  |  |  |  | 1- The radiology teaching method used made me interested in radiology: |
|  |  |  |  |  | 2- I am satisfied with the organization of radiology training in the manner used: |
|  |  |  |  |  | 3- I am satisfied with the activities during radiology training: |
|  |  |  |  |  | 4- The learning activities used are easily accepted: |
|  |  |  |  |  | 5- The radiology teaching method used has strengthened my knowledge of anatomy: |
|  |  |  |  |  | 6- Knowledge is more easily accepted by the method used for radiology education: |
|  |  |  |  |  | 7- Radiology education has increased my understanding of different imaging methods: |
|  |  |  |  |  | 8- The radiology training method has increased my confidence to face in the future: |
|  |  |  |  |  | 9- The radiology teaching method has increased my understanding of the daily clinical work in the radiology department: |
|  |  |  |  |  | 10- I am generally satisfied with the learning activities used: |

Appendix 1- Student satisfaction questionnaire:
