# Supplementary material for: Using PACS for teaching radiology to undergraduate medical students
Source: BMC Med Educ. 2024 Aug 28;24:935. doi: 10.1186/s12909-024-05919-9 (PMC11351050; doi:10.1186/s12909-024-05919-9)
Supplement: Supplementary file 2 — Supplementary Material 2 [file 12909_2024_5919_MOESM2_ESM.docx]

| I strongly disagree  (1) | I disagree  (2) | I have no opinion (3) | I agree (4) | I strongly agree (5) |  |
| --- | --- | --- | --- | --- | --- |
|  |  |  |  |  | 1- I am familiar with the capabilities of PACS: |
|  |  |  |  |  | 2- I am familiar with the principles of CT scan: |
|  |  |  |  |  | 3- I am familiar with the principles of CT interpretation: |
|  |  |  |  |  | 4- I am familiar with the principles of reconstruction methods of CT images: |
|  |  |  |  |  | 5- I fully understand how to set the center and width of the appropriate window for checking images: |
|  |  |  |  |  | 6- I am familiar with the density of different textures to choose the right center and width of the window: |
|  |  |  |  |  | 7- I am familiar with the location of different members in cross-sectional images: |
|  |  |  |  |  | 8- I am familiar with the vicinity of different organs and can reconstruct in my mind: |
|  |  |  |  |  | 9- I feel confident in the internship course to read CT: |
|  |  |  |  |  | 10- I agree that PACS is useful for learning clinical imaging: |
|  |  |  |  |  | 11- I am interested in radiology: |
|  |  |  |  |  | 12- I think I might become a radiologist: |

Appendix 2- Student self-assessment questionnaire:
